# Dietary Protein and Energy Balance in Relation to Obesity and Co-morbidities

**DOI:** 10.3389/fendo.2018.00443

**Published:** 2018-08-06

**Authors:** Mathijs Drummen, Lea Tischmann, Blandine Gatta-Cherifi, Tanja Adam, Margriet Westerterp-Plantenga

**Affiliations:** ^1^Faculty of Health, Medicine and Life Sciences, School of Nutrition and Translational Research in Metabolism (NUTRIM), Maastricht UMC+, Maastricht University, Maastricht, Netherlands; ^2^Department of Endocrinology, Diabetology and Nutrition, Universite de Bordeaux, Bordeaux, France

**Keywords:** dietary protein, energy balance, protein turnover, food-reward, obesity, NAFDL, type 2 diabetes, cardiovascular disease

## Abstract

Dietary protein is effective for body-weight management, in that it promotes satiety, energy expenditure, and changes body-composition in favor of fat-free body mass. With respect to body-weight management, the effects of diets varying in protein differ according to energy balance. During energy restriction, sustaining protein intake at the level of requirement appears to be sufficient to aid body weight loss and fat loss. An additional increase of protein intake does not induce a larger loss of body weight, but can be effective to maintain a larger amount of fat-free mass. Protein induced satiety is likely a combined expression with direct and indirect effects of elevated plasma amino acid and anorexigenic hormone concentrations, increased diet-induced thermogenesis, and ketogenic state, all feed-back on the central nervous system. The decline in energy expenditure and sleeping metabolic rate as a result of body weight loss is less on a high-protein than on a medium-protein diet. In addition, higher rates of energy expenditure have been observed as acute responses to energy-balanced high-protein diets. In energy balance, high protein diets may be beneficial to prevent the development of a positive energy balance, whereas low-protein diets may facilitate this. High protein-low carbohydrate diets may be favorable for the control of intrahepatic triglyceride IHTG in healthy humans, likely as a result of combined effects involving changes in protein and carbohydrate intake. Body weight loss and subsequent weight maintenance usually shows favorable effects in relation to insulin sensitivity, although some risks may be present. Promotion of insulin sensitivity beyond its effect on body-weight loss and subsequent body-weight maintenance seems unlikely. In conclusion, higher-protein diets may reduce overweight and obesity, yet whether high-protein diets, beyond their effect on body-weight management, contribute to prevention of increases in non-alcoholic fatty liver disease NAFLD, type 2 diabetes and cardiovascular diseases is inconclusive.

## Introduction

The prevalence of obesity and its associated co-morbidities, such as non-alcoholic fatty liver disease (NAFLD), type 2 diabetes and cardiovascular diseases, has increased in a growing number of countries (1, 2). Energy intake exceeding energy expenditure results in a chronic positive energy balance, storage of excess energy, and subsequent body weight gain (3). Treatment of obesity requires a negative energy balance, which most efficiently and effectively is achieved by applying an energy-restricted diet (4). However, this usually results in increased feelings of hunger and desire to eat, and in a decrease of the feeling of fullness, implying a risk for sustaining a lower energy intake. Body weight loss consists of loss of fat mass and of fat-free mass (FFM); the latter causes a reduction in energy expenditure and a decrease in energy requirement. This vicious cycle may counteract the negative energy balance induced by the energy-restricted diet. Consequently, body weight loss should be paralleled by a reduction in energy intake without changing appetite, and maintenance of energy expenditure by preserving FFM. Both goals can be achieved through an energy-restricted, relatively high-protein diet (5–8). In this review the mechanisms of protein-induced appetite modulation, reward homeostasis, and energy expenditure are highlighted, including possible adverse effects of protein-diets. Finally, the relevance of relatively high-protein diets for treatment or prevention of NAFLD, cardiovascular diseases and type 2 diabetes apart from weight-loss and subsequent weight maintenance are discussed.

## Short-term dietary protein-induced energy homeostasis - satiety

Short-term intervention studies using energy-balanced diets with large contrasts in relative protein content have shown that high-protein diets are more satiating than diets lower in protein (9–15). Furthermore, subjects consumed less food during an *ad libitum* high-protein diet relative to baseline (16), while being similarly satiated and satisfied (16–18) Dietary proteins exert a high satiating effect via different pathways including stimulation of gut hormone secretion, digestion effects, circulating amino-acid levels, energy expenditure, a ketogenic state, and possibly gluconeogenesis. Here the gut-brain axis, encompassing signaling from gastrointestinal hormones released in the blood and acting at their brain receptors, conducts signals to the brain deriving from the gastrointestinal system contributing to control of energy intake.

### Aminostatic theory

Elevated blood concentrations of amino acids may stimulate satiety signaling in the brain (13, 19–23). According to the “aminostatic theory,” serum amino acids that cannot be channeled into protein synthesis directly serve as satiety signals (24). However, the aminostatic theory failed to gain strong support because fasting circulating amino acid levels do not correlate with appetitive sensations and there are non-congruent appetitive responses to protein sources varying in the rate of amino acid appearance. Indirectly, dietary amino acids may act on satiety signaling via receptors in the duodeno-intestinal and hepatoportal regions (25). Depending on the type of amino acid, they increase or decrease the activity of hepatic vagal afferent fibers, innervating satiety centers in the brain (25). The branched-chain amino acids leucine, isoleucine and valine reportedly contribute to satiety, following these mechanisms (13, 19–21, 23, 25).

### Role of anorexigenic and orexigenic gut hormones

The satiety-stimulating effect of protein is partly related to increases in anorexigenic gut hormones, produced in response to peripheral and central detection of amino acids (23, 26–30). They react to elevated protein intake and stimulate vagal activity in brain areas involved in the control of food intake (23, 31, 32). Concentrations of glucagon-like peptide 1 (GLP-1), cholecystokinin (CCK), and peptide YY (PYY) consistently increase in response to high protein intakes (23, 26–30). Apart from its effect on anorexigenic hormones, protein intake can also influence orexigenic tone. Therefore, dietary protein consumed in liquid preloads prolongs the postprandial suppression of ghrelin (33, 34). This response was not affected by the type of protein consumed (soy, whey, or gluten) and was similarly observed in lean and overweight subject. The ghrelin decrease was also shown during a whey-protein infusion intraduodenally administered with a dose dependent effect (35, 36).

Cortisol response has also been studied after protein ingestion with a significant decline of serum cortisol within 30 min after amino-acid ingestion (37). This underlines the general view that amino-acids also stimulate catabolic pathways. In addition, effects of protein on the orexigenic endocannabinoids have to be investigated (38).

### Possible relations of changes in amino-acid concentrations or gut hormones with satiety

Acute amino acid-related effects on satiety, depending on the quality proteins, have been reported. The digestion of “fast” proteins, such as whey, results in high and early rises of plasma amino acids and appetite hormones (23). The slower digestion and absorption rates of casein result in more prolonged and maintained plasma amino acid and hormone concentrations than those of whey (13, 22, 39, 40). However, at high concentrations, no clear evidence exists for differences in satiating capacity between different types of protein, (11, 21–23, 28, 33, 41–43). The concentrations of certain amino acids have to be above a particular minimum threshold to promote a relatively stronger hunger suppression or greater fullness (23). Indispensible or complete proteins reach these thresholds at lower concentrations than other, dispensible or incomplete proteins. Deficiency of essential amino-acids may lead to suppression of intake of food consisting of incomplete proteins (44). A chemosensor for essential amino acid deficiency is present in the anterior periform cortex (45), signaling brain areas that control food intake (44). Likewise, consumption of an incomplete protein may be detected and result in a signal to stop eating in humans (46). The signal of incomplete proteins is rather a signal of hunger suppression than of satiation or satiety (23, 46).

### Protein-induced satiety and diet-induced thermogenesis

The relationship between protein-induced satiety and diet-induced thermogenesis, or DIT, is explained by increased energy expenditure at rest implying increases in oxygen consumption and body temperature. The feeling of oxygen deprivation is translated into satiety feelings (12, 47). A positive relationship between an increase in satiety and at the same time an increase in 24-h DIT has been observed with an energy-balanced high-protein diet (12, 48).

### High-protein low-carbohydrate induced ketogenic effect and gluconeogenesis

Fasting β-hydroxybutyrate concentrations increase in response to a ketogenic high-protein, “low-carb” diet compared with an isoenergetic medium-protein, medium-carbohydrate diet (49–51). Increased concentrations of β-hydroxybutyrate directly affected satiety in a 36 h study (52). Gluconeogenesis and satiety were increased at a zero carbohydrate, high-protein diet, however, these were unrelated to each other, yet the increased concentration of β-hydroxybutyrate contributed to satiety in the high-protein diet (53).

In general, in short-term experiments, *ad libitum* high-protein diets have been observed to sustain appetite at a level comparable to the original diet, despite a lower energy intake. Energy-restricted, high-protein diets produce a sustained lower energy intake compared to diets with lower protein content, without altered appetite and satiety scores (17, 54). Consequently, individuals who consume a high-protein diet in combination with energy-restriction are more satiated and potentially less likely to consume additional calories from foods extraneous to dietary prescription (10).

From short-term experiments we conclude that relatively high-protein diets have the potential to maintain a negative energy balance by sustaining satiety at the level of the original diet (9, 16). This strong satiety effect depends partly on the type of dietary protein, and is elicited by a mixture of gut-brain axis effects, such as anorexigenic gut hormones, digestion, amino-acids, ketogenesis, and the increase in diet-induced thermogenesis. Gluconeogenesis did not show a relation with satiety (53).

## Short-term dietary protein-induced reward homeostasis

Although dietary protein-induced satiety affects energy intake, it may be dominated by reward-driven eating behavior (20, 31, 55–57). Several brain areas that are involved in food reward link high-protein intake with reduced food wanting and thereby act as a mechanism involved in the reduced energy intake following high protein intake (20, 31, 55–57). A mechanism through which protein acts on brain reward centers involves direct effects of certain amino acids as precursors of the neuropeptides serotonin and dopamine (31, 55). A high-protein, low-carbohydrate breakfast vs. a medium-protein, high-carbohydrate breakfast led to reduced reward-related activation in the hippocampus and parahippocampus before dinner (20, 32). Furthermore, acute food-choice compensation changed the macronutrient composition of a subsequent meal to offset the protein intervention (56). A compensatory increase in carbohydrate intake was related to a decrease in liking and task-related signaling in the hypothalamus after a high-protein breakfast. After a lower-protein breakfast, an increase in wanting and task-related signaling in the hypothalamus was related to a relative increase in protein intake in a subsequent meal (56). Protein intake may directly affect the rewarding value of this macronutrient (56, 58). Thus limited protein-induced food reward may affect compliance to a long-term protein-diet.

## Short-term dietary protein-induced energy expenditure—effects and mechanisms

With respect to dietary protein-induced energy expenditure, short-term effects of energy-balanced high-protein diets showed higher rates of energy expenditure, especially diet-induced thermogenesis (DIT) (59, 60). Mechanisms encompass the ATP required for the initial steps of metabolism, such as protein breakdown, synthesis and storage, and oxidation including urea synthesis. Also gluconeogenesis may take place. Protein storage capacity of the body is limited. Therefore readily metabolic processing is necessary. The magnitude of DIT is determined by the level of energy intake in relation to energy requirement and the type of protein, and is illustrated by the difference between the gross energy value of 22–25 kJ/g and the net metabolizable energy of 13 kJ/g. DIT values for separate proteins are 20–30% of energy intake from protein (61).

Significantly higher dietary protein induced DIT (59), subsequently Sleeping Metabolic Rate (SMR) and Basal Metabolic Rate (BMR) (12, 60) was shown in 36 h respiration chamber studies, in comparison to iso-energetic, iso-volumetric, dietary carbohydrate, or fat, composed of normal food items and matched organoleptic properties. Short-term protein- induced increase in DIT is explained by the ATP required for the initial steps of metabolism and oxidation including urea synthesis, while subsequent protein induced increase of SMR is explained by stimulation of protein synthesis and protein turnover. The metabolic efficacy of protein oxidation largely depends on the amino acid composition of the protein (62). A well-balanced amino acid mixture produces a higher thermogenic response than does an amino acid mixture with a lower biological value, explaining why intake of plant proteins or incomplete proteins results in less protein synthesis than does intake of animal protein. Based upon the stoichiometry of amino acid catabolism and urea synthesis, the calculated energy expenditure to produce ATP is ranging from 153 kJ/ATP for cysteine, to 99 kJ/ATP for glutamate (63). This relative metabolic inefficiency contributes to the higher diet-induced energy expenditure of a high protein meal, which, in turn, has shown to be related to subjective feelings of satiety (48). Gluconeogenesis, as a result of further postprandial amino-acid metabolism also contributes to the protein induced energy expenditure. *De novo* synthesis of glucose in the liver from gluconeogenic precursors including amino acids is stimulated by a high protein diet in the fed state (64, 65), and is an alternative biochemical pathway to cope with postprandial amino acid excess (66). When the protein content of the diet is increased, Phosphoenolpyruvate Carboxylase (PEPCK) that catalyzes the initial conversion of oxaloacetate to phosphoenolpyruvate is up-regulated either in the fasted and in the fed state, whereas glucose 6-phosphatase (G6Pase), that catalyzes the last step of gluconeogenesis is up-regulated in the fasted state and down-regulated in the fed state (64). Although hepatic glycogen stores as well as hepatic gluconeogenesis have been suggested to play a role in the regulation of satiety (67, 68), this was not confirmed by a study by Veldhorst et al. (49) However, they showed that gluconeogenesis strongly increased energy expenditure, in that 42% of the increase in energy expenditure after the high-protein diet was explained by the increase in gluconeogenesis. The cost of gluconeogenesis was 33% of the energy content of the produced glucose (49).

### Protein turnover, protein breakdown, and protein synthesis

Also protein turnover contributes to the high energetic costs of protein metabolism, and protein synthesis. The daily protein turnover of a healthy adult - defined as synthesis plus breakdown-, 300 gram/day, depends on the type of protein, and age. It is high in children, and decreases with older age. Increasing protein intake increases protein turnover by increasing protein synthesis and protein breakdown, and does not necessarily affect protein balance (69, 70). Rapidly digested dietary protein results in a stronger increase in postprandial protein synthesis and amino acid oxidation than slowly digested protein (39, 40, 71).

Acutely, high protein intake stimulates protein synthesis and turnover, and induces a small suppression of protein breakdown (72–74). Prolonged low protein intake may lead to muscle loss due to the lack of precursor amino acid availability for *de novo* muscle protein synthesis (75, 76). Hursel et al. (70) observed that protein turnover was significantly higher after a 12-week high-protein vs. low-protein diet, with significant increases in protein synthesis, protein breakdown, and protein oxidation. Notably, in the fasted state net protein balance was less negative after the low-protein diet compared with the high-protein diet, while in the fed state, protein balance was positive with the high-protein diet, and negative with the low-protein diet (70). Thus protein turnover in the fasted state needs to be distinguished from that in the fed state. The role of protein synthesis and protein breakdown in FFM accretion was discussed by Deutz and Wolfe (77), and Symons et al. (76). The observed maximum response of protein synthesis after a single serving of 20–30 g of dietary protein suggests that additional effects of protein intake on FFM accretion are accounted for by the inhibition of protein breakdown. However, a beneficial reduction of protein breakdown only occurs with acute ingestion of protein (70, 76, 78–80). The positive protein balance observed at a high-protein diet is due to acute postprandial responses, rather than to the postabsorptive state.

Consumption of a low-protein diet for 12 weeks was not detrimental to young healthy individuals who might have the ability to adapt acutely to this condition (70). The Adaptive Demands model developed by Millward may provide an explanation for the observation that the human body is able to show physiological adaptations to changes in protein intake (81). The model proposes that the metabolic demand for amino acids comprises a fixed component and a variable adaptive component (81). Short-term changes in protein intake are likely within the adaptive range. Adaptations in protein and amino acid metabolism to changes in protein intake largely occur via changes in whole-body protein turnover and amino acid oxidation (82). Changes in amino acid oxidation were reflected as decreased and increased nitrogen excretion in response to the low- and high-protein diets respectively. The activity of enzymes that regulate: (1) transamination, (2) the disposal of the carbon skeletons in intermediary metabolism, and (3) the disposal of nitrogen through the urea cycle increased in response to high protein intake (83, 84). Nevertheless, a positive nitrogen balance following high protein intake (69, 82, 85, 86) does not automatically reflect an increase in protein anabolism (87). The capacity of the body to increase amino acid anabolism through an increase in lean body mass is limited (87). Only interventions using diets high in specific indispensable amino acids, such as leucine, might be able to stimulate protein synthesis in specific target groups (73, 88). Therefore, transient retention or loss of body nitrogen because of a labile pool of body nitrogen may contribute to adaptations in amino acid metabolism in response to changes in protein intake (89). Transient adaptive mechanisms may be distinguished from mechanisms that maintain homeostasis in the body in the longer-term.

## Long-term dietary protein effects during energy restriction, body-weight loss, and body-weight maintenance

Most long-term studies comparing energy-restricted diets with a relatively high protein content and diets with a normal protein content, within a large range of fat contents, showed independent effects of a high protein intake on body weight reduction (7, 90–93, 93–110), while in other studies the opposite has been observed (111–121). From these studies, a larger reduction in fat mass following relatively high-protein diets was reported by Wycherly et al. (7), Soenen et al. (90), Brinkworth et al. (93), Brinkworth et al. (94), Due et al. (96), Gardner et al. (98), Jesudason et al. (101), Layman et al. (102), Das et al. (112), Foster et al. (115, 122), Frisch et al. (97), Brinkworth et al. (123), Keogh et al. (121), Krebs et al. (104, 119), while less reduction in fat mass was reported by Clifton et al. (113). An energy-restricted high-protein diet in combination with exercise can even increase muscle mass (124). The main reason behind the differences in outcomes of the studies cited, is the difference in dosage of dietary protein (125). If the control, implying an adequate protein intake is sufficiently high, i.e., 0.66 g/kg body weight daily, then no differences in body weight effects are expected. In case the relatively high protein diet is higher than 1.2 g/kg body weight daily, then a fat free mass sparing effect can be expected.

Conclusions from long-term studies comparing relatively high-protein with normal-protein diets differ from those testing relatively high-protein and low-protein diets (5). In the following studies compliance was monitored and confirmed with a quantitative biomarker, such as urinary nitrogen (5, 7, 91). A relatively high protein diet (in % of energy) implies a restriction in carbohydrate and fat intake, but no restriction of protein intake (in g/d), thus a protein intake comparable to the original diet. During energy restriction, sustaining protein intake at the level of the minimal requirement (0.66 g/kg body weight daily) appears not to hinder body weight loss and fat loss (7, 91, 126). An additional increase of protein intake may not induce a larger loss of body weight, but can be effective to maintain a larger amount of FFM (7, 91, 126) and limits the reduction of energy expenditure through sparing of FFM (91, 127). For example, a 6-month energy restricted diet with a daily protein intake just above the minimal requirement (0.8 g/kg body weight daily) induced a comparable reduction in body weight to an energy-restricted diet with a daily protein intake well above the minimal requirement (1.2 g/kg body weight daily) (91). Interestingly, a protein intake of 1.2 g/kg body weight daily resulted in a stronger decrease in fat mass and preservation of FFM (91). Dietary protein intake below requirements could lead to less weight loss and a higher risk for body weight regain (6). Increasing the relative protein content of a diet automatically results in a decrease in the relative content of carbohydrate and/or fat, which theoretically could be a factor in triggering the described effects. However, a study by Soenen et al. demonstrated that the effects of a relatively high protein intake on body weight loss and weight maintenance were present independent of a low carbohydrate intake (90), and that low carbohydrate alone, without high protein did not trigger the described effects.

Protein diets could have resulted in stronger effects with respect to body weight management, if compliance would have been larger (see section Short-term Dietary Protein-Induced Reward Homeostasis). To counteract poor compliance, dietary restraint is necessary (128). In several long term clinical trials with dietary protein, cognitive dietary restraint had increased, implying greater conscious control over food intake (68, 90, 91). *Post hoc* analysis of those data shows that the change in the cognitive dietary restraint score was inversely related to the change in body weight. Dietary restraint is associated with brain signaling for reward, indicating a greater control over food intake and implying a greater control over reward as well (129). In general dietary restraint is associated with long-term weight maintenance (130, 131).

Taken together, Clifton et al. (125) conclude from a recent meta-analysis that the short-term benefit of higher protein diets persists to a small degree over the long term, depending on dietary compliance.

### Body composition

Older studies, in the perspective of composing meal replacers to be used as energy restricted diets showed strong energy restriction effects on body composition, in relation to the percentage from dietary protein. The protein content of a formula diet was varied from 0 to 50 g/d resulting in a protein loss of between 1202 and 91 grams, respectively, over 28 days (132). Loss of fat mass (FM) as a percentage of body weight loss was 43% with 0 g/d protein, and up to 79% with 50 g/d protein, indicating a change in body composition including sparing of fat free mass (FFM), due to the amount of protein intake. Similarly, during weight maintenance following weight loss, FFM was preserved, while FM was reduced. Since weight maintenance after weight loss usually implies a slight weight regain, Stock's model can be applied (133). The greatest metabolic efficiency of weight gain is shown when protein intake is 10–15% of energy and inefficiency is shown with < 5 and >20% of energy from protein. The latter metabolic inefficiency is related to body composition. For 1 kg of body mass with 60% FM and 40% FFM, an additional 30 MJ needs to be ingested, whereas for 1 kg of only FFM, an additional 50–70 MJ is needed (133, 134). This metabolic inefficiency, partly due to sparing FFM promotes dietary protein induced weight maintenance. In addition, preserving FFM, being the main determinant of basal energy expenditure, limits a possible reduction in energy expenditure during weight maintenance. Whitehead et al. showed that during energy-restriction, the decline in total energy expenditure and SMR as a result of body weight loss is less on a high-protein than on a medium-protein diet (135). Even an increase in FFM during a high-protein diet in negative energy balance has been observed (124), although these changes may partly be ascribed to a high protein intake combined with physical activity.

## Prevention of overweight: role of dietary protein in neutral energy balance

If the protein-induced effects on appetite and energy expenditure observed during energy restriction also hold under non-restricted conditions, then, increasing protein intake with a usual diet may prevent overweight and obesity. A 12-week intervention study was performed comparing high-protein (30% of energy from protein) and low-protein (5% of energy from protein) diets, in weight stable individuals (136). In this controlled situation, participants were able to sustain the high- and low-protein diets. The low-protein diet facilitated the development of positive energy balance, while the high-protein diet was beneficial to prevent this (136). Correspondingly, small increases in fullness and satiety ratings were observed as acute responses to a high-protein diet in neutral energy balance (136). In this situation, translation into large changes in energy intake was not possible, because subjects had to maintain their body weight. In the longer term, appetite ratings were returned to the level of the original diet, which suggests that the human body habituates to the satiating effects of high protein intake (136). FFM showed small increases and decreases after a 12-week intervention with high-protein and low-protein diets in energy balance (136). As a consequence, SMR, DIT, and total energy expenditure was maintained at the high-protein diet, while it was significantly decreased at the low protein diet. Thus, at a constant body weight, a high-protein diet may protect against the development of a positive energy balance. The consumption of a low-protein diet may increase the risk for the development of a positive energy balance through adaptive thermogenesis (136).

## Dietary protein and overweight related co-morbidities

Humans with overweight or obesity may show co-morbidities, such as a nonalcoholic fatty liver disease, type 2 diabetes, or cardiovascular diseases. Whether a high-protein diet may be protective against these co-morbidities, independent of, or in addition to effects of weight loss is still under debate.

### Nonalcoholic fatty liver disease (NAFLD)

In general, weight loss improves metabolic function (6, 7), yet a high protein intake may modulate intrahepatic triglyceride (IHTG) content as well (137–139). In short-term studies, protein supplementation was shown to be associated with reduced hepatic fat (137–139). High ectopic lipid content, especially IHTG content, and not visceral adipose tissue (VAT) volume, is an independent risk factor for these metabolic disturbances (140–142). A 12-week intervention study showed that effects of high- and low-protein diets on IHTG content in weight-stable individuals tended to lower IHTG content after the high protein-low carbohydrate diet compared with the low protein-high carbohydrate diet (127). This suggests that high protein-low carbohydrate diets may be favorable for the control of IHTG in healthy humans. High protein intake stimulates hepatic lipid oxidation due to the high energetic demand for amino acid catabolism and ketogenesis (5, 49). Furthermore, hepatic lipid oxidation may be stimulated by an increased bile acid production, a process that may also inhibit lipogenesis (143). Protein-induced glucagon secretion inhibits *de novo* lipogenesis and stimulates hepatic ketogenesis (144, 145). High protein intake may blunt the increase of very low density lipoprotein (VLDL)-TG concentrations induced by carbohydrate intake (146–148). High VLDL-TG concentrations may increase hepatic TG, and subsequently IHTG content (148). The observed trend for a difference in IHTG content between the diets likely is the result of combined effects involving changes in protein and carbohydrate intake.

### Type 2 diabetes

Relevant diets possibly contributing to the management of type 2 diabetes are low-carbohydrate diets. Those diets often are high-protein diets. A recent systematic review explored the interpretation and effectiveness of a low-carbohydrate diet in the management of type 2 diabetes (149). They suggest that low-carbohydrate diets may improve HbA1c, HDL cholesterol, and triglyceride levels. The meta-analyses confirmed statistically significant superiority of the low-carbohydrate intervention arm in improving HbA1c, HDL cholesterol, triglyceride, and systolic blood pressure levels at 1 year. Reducing carbohydrate intake demonstrated a strong superiority over control diets in reducing diabetes medication, which may have diminished the observed effects of a reduced-carbohydrate intake on HbA1c. This review concludes that reducing carbohydrate intake may promote favorable health outcomes in the management of type 2 diabetes in the context of a healthy diet (149). The relation between high-protein intake and type 2 diabetes is still under debate, and results differ depending on study duration and source of protein. Short-term studies have reported favorable effects on glucose homeostasis (21, 150, 151), while an epidemiological and a long-term studies reported an increased risk for type 2 diabetes with increased protein intake (152–154). The increased risk may be dependent on the source of protein. Tian et al. conducted a systematic review and meta-analysis of cohort studies to investigate the association between protein consumption and the risk for type 2 diabetes (155). In this review, they reported an increased relative risk of type 2 diabetes for total protein and animal protein in men and women and a reduced relative risk for plant protein in women.

However, high-protein diets may have some risk regarding insulin sensitivity. An increase in Branched-Chain Amino Acids (BCAAs) seems to be a marker of type 2 diabetes (156, 157). Newgard et al. observed in rodents that in the context of a dietary pattern that includes high fat consumption BCAA contributes to the development of obesity-associated insulin resistance. Moreover, Pedersen et al. (158) showed that the serum metabolome of insulin-resistant individuals is characterized by increased levels of BCAAs, which correlate with a human gut microbiome that has an enriched biosynthetic potential for BCAA.

Taken together, when protein diets are applied during energy restriction aiming at weight loss and subsequent weight maintenance, the latter usually shows favorable effects in relation to insulin sensitivity, although some risks may be present. That a higher protein diet would promote insulin sensitivity beyond its effect on body-weight loss and subsequent body-weight maintenance seems unlikely.

### Cardiovascular diseases

Parameters that indicate cardiovascular risks usually change in a favorable direction during body weight loss. The question remains whether the type of diet, especially a protein diet, would affect favorable changes in cardiovascular parameters. Atherosclerosis lies at the root of cardiovascular complications, and the main indicators are the HDL- and LDL cholesterol. Certain proteins may exert a greater effect on blood cholesterol levels than other (159). Possible different effects from vegetable vs. animal proteins have been tested. Sacks et al. (160) compared diets enriched in casein for 20 days with diets enriched in soy for 20 days. They did not observe significant differences in LDL or HDL cholesterol, neither between lipid profiles or lipid proteins. Other studies, comparing casein and soy diets, did find significant reductions in LDL with the soy diet, compared to the casein diets (161, 162), however this did not appear in volunteers with already high cholesterol concentrations (163). In long term weight loss and subsequent weight maintenance studies, it was shown that individuals consuming soy meal replacements showed favorable effects in their cardiovascular profile, e.g., lowering LDL, TG, visceral fat, and systolic blood pressure (164). With respect to blood pressure, a study by Teunissen-Beekman et al. (165) compared postprandial blood pressure-related responses to the ingestion of pea protein, milk protein, and egg-white protein. They concluded that lower postprandial blood pressure is not necessarily accompanied by higher NOx, insulin, glucagon or GLP-1 responses, and that dietary protein, especially egg-white protein, may induce a risk for elevated blood pressure (165). Yet, it has been reported that effects of dietary protein depend on age. Tielemans et al. (166), showed that intake of plant protein, but not animal protein, was inversely associated with 5-year changes in blood pressure level in elderly men. A critical evaluation of the evidence for the effects of milk proteins and their associated peptides on blood pressure and vascular dysfunction, showed that results are inconclusive, while one study clearly reported that main intact milk proteins reduced blood pressure, and whey protein improved measures of arterial stiffness (167). Some epidemiological studies based upon large community cohorts report no overall relationship between protein type and dietary protein sources on coronary heart diseases (168), while another epidemiological study indicated that high red meat intake increases risk for coronary heart disease and stroke, and that poultry, fish, and nuts reduced these risks (169, 170). A general systematic review on health effects of protein intake in healthy adults reported that results are inconclusive for a relationship between protein intake and cardiovascular diseases (171), while a recent systematic review concluded that low-carbohydrate diets, that often are high-protein diets may improve HDL cholesterol and triglyceride levels and systolic blood pressure levels at 1 year (149). Taken together, more accurately designed randomized control trials on dietary protein quality and quantity and possible relations with cardiovascular risks are required.

## Adverse effects of protein-diets

There is a long-held view that high-protein intake might interfere with calcium homeostasis by increasing the acid load. It is hypothesized that this could be partially buffered by bone, subsequently resulting in bone resorption and hypercalciuria (172). In general, protein is a necessary nutrient for bone health (173). Nitrogen intake seems to have a positive effect on calcium balance and consequent preservation of bone mineral content (174). With respect to renal issues, only patients with pre-existing dysfunction appeared to have an increased risk for the development of kidney stones and renal diseases (172). In addition, Jesudason et al. (101) showed that both a medium or higher protein energy restriction diet, inducing body weight loss, normalized renal function in individuals with hyperfiltration. Similarly, (175) showed in a study in volunteers with overweight or obesity and pre-diabetes on a higher protein diet, a significant increase in urinary urea/creatinine ratio and serum urea after 1 year. There were no associations between increased protein intake and creatinine clearance, estimated glomerular filtration rate, urinary albumin/creatinine ratio, or serum creatinine. They found no indication of impaired kidney function after 1 year with a higher protein intake in pre-diabetic older adults. In the elderly, beneficial health effects of higher-protein intake might outweigh the adverse effects possibly because of the changes in protein metabolism with aging. In contrast, persistent total protein and amino acid intake below requirements impairs bodily functions leading to higher disease and mortality risks across the lifespan (176, 177). Taken together, application of relatively high-protein diets, whereby protein intake is sustained at the original level, does not seem to have any adverse effects in healthy individuals. Although no clear recommendation exists that defines the safe upper limit of protein intake, consumption of up to 1.66 g/kg BW daily has not been associated with increased health risks (87, 122). This means that sustaining or slightly increasing protein intake during energy restriction likely poses no adverse effects in healthy individuals. However, protein intake can exceed the suggested safe upper limit. The question arises whether and how and over which time-frame these high intakes of protein would negatively affect health. Recent studies applying medium-term, high-protein interventions in neutral or positive energy balance did not report any adverse effects (136, 178). However, the limits of adaptation to high protein intake over the longer term remain to be investigated.

## Discussion

**Figure 1:**
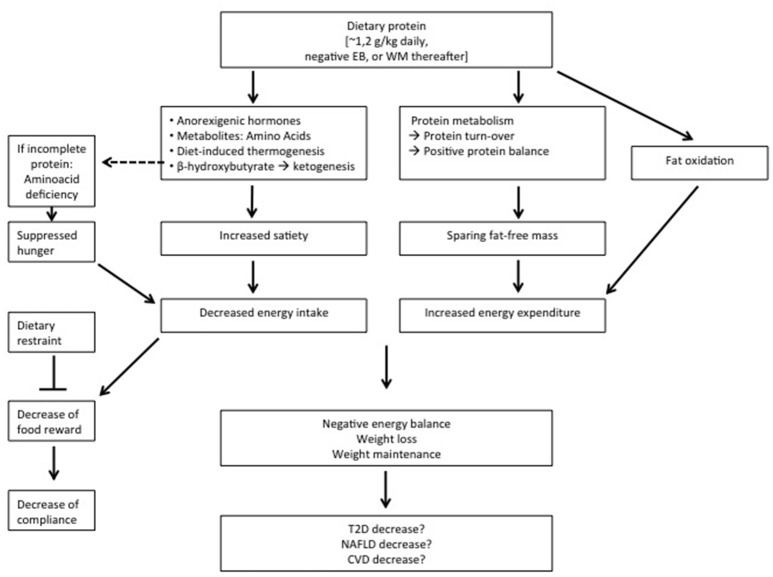
Summary of the observations on relatively high protein diets applied during energy restriction or weight maintenance (WM) thereafter. EB, energy balance; T2D, type 2 Diabetes; NAFLD, non-alcoholic fatty liver disease; CV, cardiovascular diseases.

With respect to body-weight management, the effects of diets varying in protein differ according to energy balance. During energy restriction, sustaining protein intake at the level of requirement appears to be sufficient to aid body weight loss and fat loss (Figure 1). An additional increase of protein intake does not induce a larger loss of body weight, but can be effective to maintain a larger amount of FFM (Figure 1). Protein induced satiety is likely a combined expression with direct and indirect effects of elevated plasma amino acid and anorexigenic hormone concentrations, increased DIT, and a ketogenic state, which all feed-back on the central nervous system (Figure 1). Changes in appetite appear most clearly as short-term response to changes in dietary protein content; the human body may habituate to the satiating effects of protein intake in the longer-term. The decline in energy expenditure and sleeping metabolic rate as a result of body weight loss is less on a high-protein than on a normal-protein diet. In addition, higher rates of energy expenditure have been observed as acute responses to energy-balanced high-protein diets (Figure 1). In energy balance, high protein diets may be beneficial to prevent the development of a positive energy balance, whereas low-protein diets may facilitate this. Furthermore, high protein, low carbohydrate diets may be favorable for the prevention of metabolic disturbances. During positive energy balance, excess energy intake alone may account for the increase in fat mass. Increases in energy expenditure and FFM may largely be predicted by protein intake.

High protein-low carbohydrate diets may be favorable for the control of IHTG in healthy humans, likely as a result of combined effects involving changes in protein and carbohydrate intake. When protein diets are applied during energy restriction aiming at weight loss and subsequent weight maintenance, the latter usually shows favorable effects in relation to insulin sensitivity, although some risks may be present. That a higher protein diet would promote insulin sensitivity beyond its effect on body-weight loss and subsequent body-weight maintenance seems unlikely.

At least high-protein diets do not seem to have adverse effects on these co-morbidities. In conclusion, higher-protein diets may reduce overweight and obesity, yet whether high-protein diets, beyond their effect on body-weight management, contribute to prevention of increases in NAFLD, type 2 diabetes and cardiovascular diseases is inconclusive (Figure 1).

## Author contributions

The sections of the manuscript were written by MD, LT, BG-C, TA, and MW-P. The review is partly an update of Westerterp-Plantenga et al. (4) and Westerterp-Plantenga et al. (5).

### Conflict of interest statement

The authors declare that the research was conducted in the absence of any commercial or financial relationships that could be construed as a potential conflict of interest.
